# A rapid flow strategy for the oxidative cyanation of secondary and tertiary amines *via* C-H activation

**DOI:** 10.1038/s41598-017-16410-5

**Published:** 2017-11-24

**Authors:** Kidus Tadele, Sanny Verma, Mallikarjuna N. Nadagouda, Michael A. Gonzalez, Rajender S. Varma

**Affiliations:** 1 Oak Ridge Institute for Science and Education, P. O. Box 117, Oak Ridge, TN 37831 USA; 2 Water Systems Division, Water Resources Recovery Branch, National Risk Management Research Laboratory, U. S. Environmental Protection Agency, 26 West Martin Luther King Drive, MS 443, Cincinnati, Ohio 45268 USA; 3 Land and Materials Management Division, Emerging Chemistry and Engineering Branch, National Risk Management Research Laboratory, U. S. Environmental Protection Agency, 26 West Martin Luther King Drive, MS 483, Cincinnati, Ohio 45268 USA

## Abstract

An efficient continuous flow protocol has been developed for bond C-H activation which promotes the α-cyanation of secondary and tertiary amines using magnetic nano-ferrites.

## Introduction

The development of environmentally mild methods for the synthesis of materials and general transformations have been receiving tremendous attention in recent years^1–3^, that entails reducing source pollution and hazardous waste generation by the chemical industry^4,5^. Additionally, with the increasing world population and the associated upsurge in the demand for materials, the need for chemical production will only swell^6^. Therefore, the development of clean, sustainable and environmental-friendly methods for the production of various chemical products is of the utmost importance^7^. Importantly, the elaboration of newer technologies and advancements in chemical processing can have added sustenance^8^. Engaged in the development of more sustainable protocols^9–13^, herein, we demonstrate the use of a simple continuous flow setup in presence of magnetic nano-ferrites (Fe_3_O_4_) for the direct oxidative cyanation of the C-H bond comprising secondary and tertiary amines.

The product of the direct oxidative cyanation of the C-H bond in secondary and tertiary amines have vast applications^14^ as attested by recent research efforts in this area^15–19^. This direct oxidative cyanation of the C-H bond in tertiary amines to generate the corresponding α-aminonitriles has been widely studied^20,21^, in view of their applications in natural products and nitrogen containing bioactive compounds, namely alkaloids^22–24^.

An array of metal-based catalytic procedures has been reported for the direct oxidative cyanation of tertiary amines^15–21^. Although they lack much to be desired in terms of environmental and economic aspects; most reactions require the use of acetic acid^15,17–21,25^. This adds more energy and time requirements, as well as increased waste generation. Newer reports entail the direct utilization of C-H bonds using metals to circumvent the multi-step synthetic procedure. Recently, Varma and co-workers have reported the C-H activation and cyanation of amines using in-house synthesized magnetic graphitic carbon nitride (Fe@g-C_3_N_4_)^25^. Various amines were exploited using Fe@g-C_3_N_4_ under optimized conditions to produce α-aminonitriles in high yield within a reasonable time period. Significantly, the Fe@g-C_3_N_4_ catalyst could be recovered and reused repeatedly simply using an external magnet.

This paper demonstrates the utility of magnetic nano-ferrites (Fe_3_O_4_) under continuous flow conditions to conduct the cyanation of amines within a few minutes (<10 min) in high yields; enhanced ability to quickly mix the starting materials promotes the faster reaction, quicker optimization, and the formation of selective and desired products under continuous flow conditions. Consequently, various intermediates with diverse potential applications can be synthesized easily and rapidly thus rendering the approach much more advantageous when compared to traditional one-pot synthesis.

## Synthesis and characterization of catalyst

**Figure 1:**
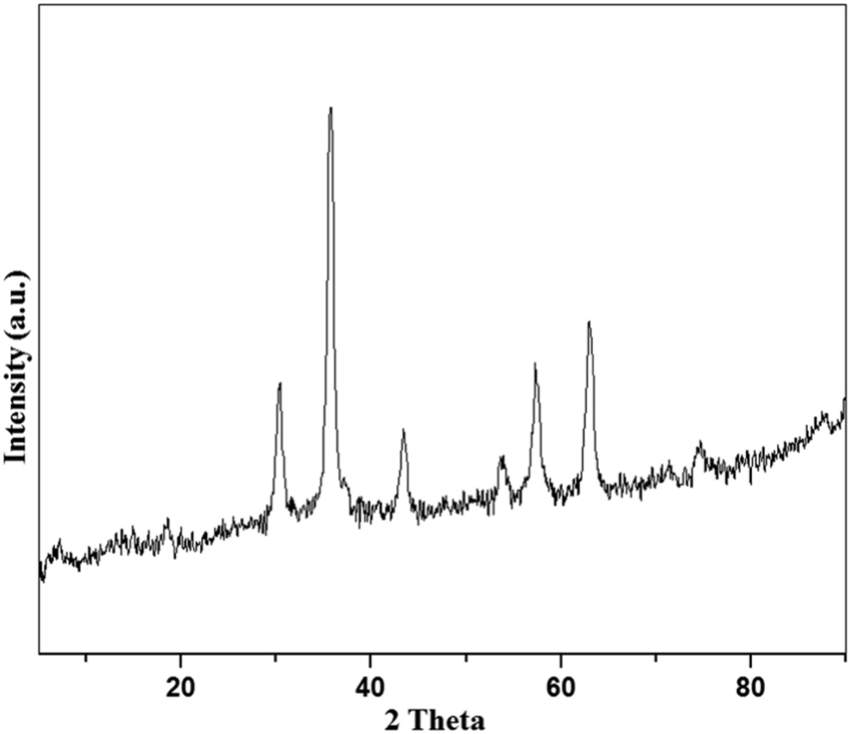
XRD analysis of magnetic nano-ferrites.

**Figure 2:**
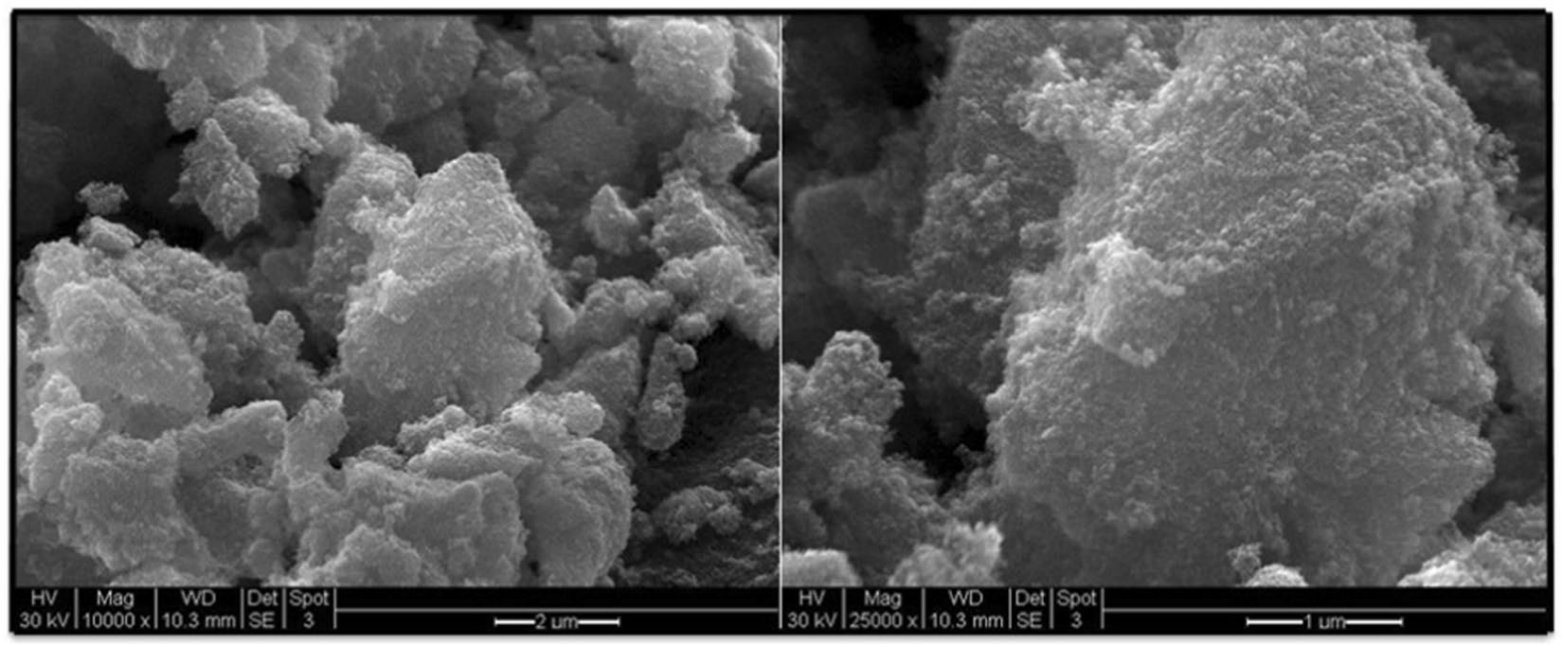
SEM analysis of magnetic nano-ferrites.

Magnetic nano-ferrites (Fe_3_O_4_) were synthesized according to reported methods^26^ and further characterized by X-ray powder diffraction (XRD), scanning electron microscope (SEM), energy dispersive X-ray analysis (EDX) and X-ray photoelectron spectroscopy (XPS). The XRD (Fig. 1) and SEM (Fig. 2) confirmed the formation of single-phase Fe_3_O_4_ nanoparticles. The presence of iron further supported by XPS (Figure S2) and EDX (Figure S3).

## Results and Discussion

The magnetic nano-ferrites (Fe_3_O_4_) were then employed for C-H bond activation and resulting cyanation of amines in a 1/16″ (i.d. 0.8mm and 10m in length) stainless steel coiled tube flow reactor (5.03mL total internal dead volume). The coil reactor is totally immersed in a Paratherm^®^ NF mineral oil bath. The oil bath was set upon a magnetic hot plate and continuously stirred to maintain a uniform temperature. The temperature attained by the oil bath facilitates efficient heating of the coiled reactor allowing transfer of heat *via* a thin film of reaction mixture flowing within the coiled tube. This allows for the reaction mixture (i.e., thin-film), coupled with the nano-ferrite catalyst, to rapidly attain the needed the reaction’s activation energy. The reaction mixture was pumped through the pre-heated coil *via* the inlet port using a peristaltic pump. This facilitated not only the lateral movement of the reaction mixture within the heated reaction zone, but also creates a consistent and well-mixed reaction fluid within the reactor. The reaction output was then collected at the exit port.

**Figure 3:**
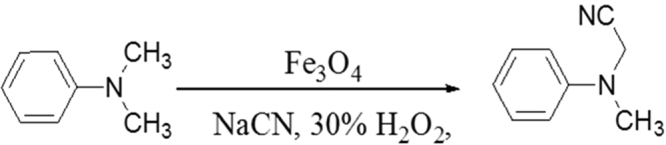
Synthesis of α-aminonitrile.

Several experimental trials for the cyanation of *N, N*-dimethylaniline were performed to establish optimized reaction conditions. All reactions were conducted in the presence of magnetic nano-ferrites (Fe_3_O_4_) by varying temperature and flow rate respectively (Table 1, entries 1–15). A reaction mixture was first prepared by dissolving *N*, *N*-dimethylaniline in a water and methanol solution (1:1 ratio). Further, 25mg of Fe_3_O_4_ catalyst, NaCN (1.1 mmol) and 30% (aq) hydrogen peroxide (1 mmol) were added (Fig. 3). This mixture was then pumped through the coil reactor at room temperature and ambient pressure. Several reactions were performed at the room temperature while varying the flow rate (and subsequently the residence time) (Table 1, entries 1–8). At flow rate of 0.9 mL min^−1^ and 1.0 mL min^−1^ (5.6 and 5.0 min residence time, respectively) traces of desired product were obtained. Decreasing the flow rate to 0.8 mL min^−1^ increased the conversion (Table 1, entry 7) however, further decrease to 0.7 mL min^−1^ did not make any difference (Table 1, entry 8). Therefore, subsequent reactions were conducted by keeping the flow rate at 0.8 mL min^−1^ and changing the temperature (Table 1, entries 9–12). Full conversion to the desired product was discernible at 50 °C and 0.8 mL min^−1^ (6.2 min residence time) (Table 1, entry 12); further increments in the flow rate while keeping the temperature at 50 °C reduced the conversion (Table 1, entries 13–15).

**Table 1:** Reaction parameter optimization for the synthesis of α-aminonitrile.

Entry	Residence time (min)	Temperature (°C)	Flow rate (mL/min)	Conversion^a^	Yield^b^
1	1.6	30	3	—	—
2	2.0	30	2.5	—	—
3	2.5	30	2.0	—	—
4	3.3	30	1.5	—	—
5	5	30	1	Trace	—
6	5.6	30	0.9	Trace	—
7	6.2	30	0.8	5%	3%
8	7.2	30	0.7	5%	3%
9	6.2	35	0.8	36%	32%
10	6.2	40	0.8	69%	68%
11	6.2	45	0.8	83%	80%
12	6.2	50	0.8	99%	97%
13	5.6	50	0.9	95%	92%
14	5.0	50	1.0	83%	80%
15	3.3	50	1.5	66%	64%
16^c^	6.2	50	0.8	—	—

^a^Reaction condition: Amine (1 mmol), 30% H_2_O_2_ (1 mmol), NaCN (1.1 mmol), magnetic nano-ferrites (25 mg), water (5 mL), methanol (5 mL); ^b^Isolated yield; ^c^In the absence of 30% H_2_O_2_.

Upon optimizing the reaction conditions, the scope of the reaction was explored using a variety of tertiary and secondary amines (Table 2, entries 1–6). Importantly, the presence of an electron withdrawing (e.g. bromo group on ortho- and para-position; Table 2, entries 2–3) and an electron donating group (e.g. methyl group on ortho- and para-position; Table 2, entries 4–5) did not affect the reaction rate and delivered the full conversion to desired products.

**Table 2:** Magnetic nano-ferrites catalyzed synthesis of α-aminonitriles *via* C-H activation.

Entry	Reactant	Product	Conversion^a^	Yield^b^
1			99	97%
2			99	95%
3			99	95%
4			99	95%
5			99	95%
6			99	94%

^a^Reaction condition: Amine (1 mmol), 30% H_2_O_2_ (1 mmol), NaCN (1.1 mmol), magnetic nano-ferrites (25 mg), water (5 mL), Methanol (5 mL); ^b^Isolated yield.

**Figure 4:**
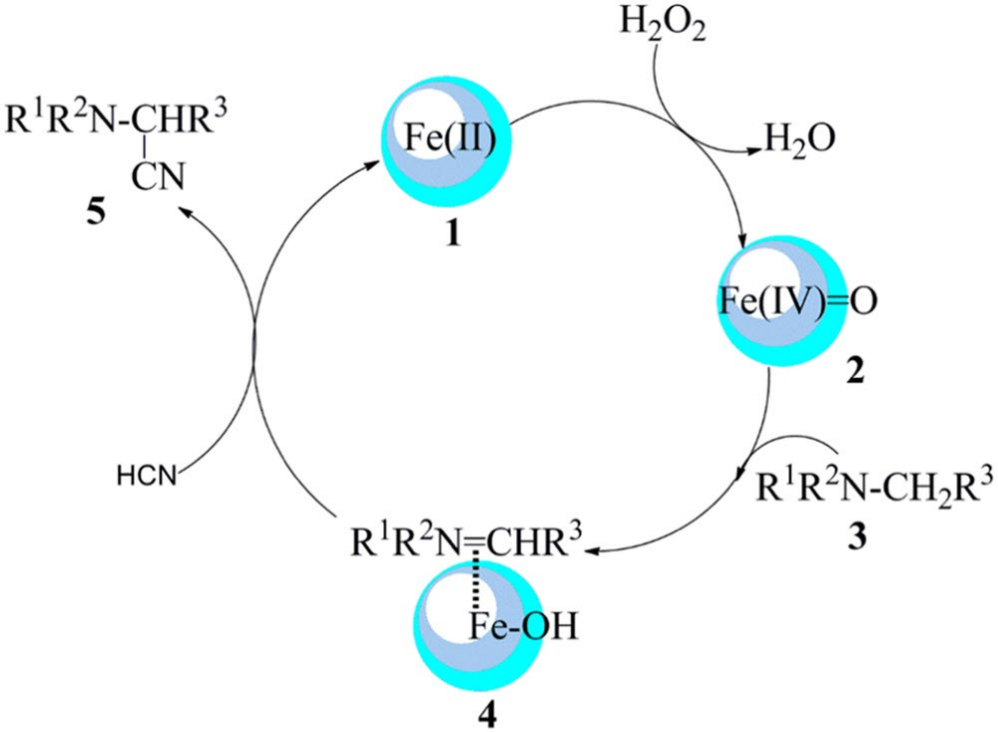
Plausible mechanism for the oxidative cyanation of amines.

A plausible mechanism has been proposed for the oxidative cyanation of amines wherein the reaction follows an oxidative and reductive mechanism. Iron (II), **1**, reacts with H_2_O_2_ leading to the formation of reactive oxo-iron (IV) species, **2** which subsequently reacts with a tertiary amine to give an iminium ion, **4**. This intermediate, **4**, reacts with *in-situ* generated HCN and delivers the corresponding α-aminonitrile (Fig. 4)^19^.

## Conclusions

Magnetic nano-ferrites coupled with a continuous flow reactor provides a protocol that has been used to arrive at a more sustainable approach for the synthesis of α-aminonitriles *via* C-H activation. This efficient method generates the desired products in less than 10 min of reaction. This strategy offers major improvements over previously reported methods which often require longer reaction times and higher temperatures rendering it more attractive in term of efficiency and ease of synthesis. Moreover, this approach is further simplified as the nano-ferrites can be easily separated using external magnet upon completion of the reaction. The recycled nano-ferrites can then be reused again without any demonstrated loss of its catalytic activity (Supplementary Information). Therefore, qualitatively speaking in terms of costs and energy, the developed protocol is more appealing and is an improved alternative over the conventional methods for the C-H activation.

### Disclaimer

The views expressed in this article are those of the authors and do not necessarily represent the views or policies of the U.S. Environmental Protection Agency. Any mention of trade names or commercial products does not constitute endorsement or recommendation for use.

## Electronic supplementary material

Supplementary Information
