# Supplementary material for: A rapid flow strategy for the oxidative cyanation of secondary and tertiary amines via C-H activation
Source: Sci Rep. 2017 Nov 24;7:16311. doi: 10.1038/s41598-017-16410-5 (PMC5701117; doi:10.1038/s41598-017-16410-5)
Supplement: Supplementary file 1 — Supplementary Information [file 41598_2017_16410_MOESM1_ESM.doc]

Supplementary Information

A rapid flow strategy for the oxidative cyanation of secondary and tertiary amines *via* C-H activation

Kidus Tadele^a^, Sanny Verma^a^, Mallikarjuna N. Nadagouda^b^ Michael A. Gonzalez^c*^and Rajender S. Varma^b^*

^a^Oak Ridge Institute for Science and Education, P. O. Box 117, Oak Ridge TN, 37831, USA.

^b^Water Systems Division, Water Resources Recovery Branch, National Risk Management Research Laboratory, U. S. Environmental Protection Agency, 26 West Martin Luther King Drive, MS 443, Cincinnati, Ohio 45268, USA.

^c^Land and Materials Management Division, Emerging Chemistry and Engineering Branch National Risk Management Research Laboratory, U. S. Environmental Protection Agency, 26 West Martin Luther King Drive, MS 483, Cincinnati, Ohio 45268, USA

**Contents**

General procedure for the synthesis of magnetic nano-ferrites

General procedure for the α-cyanation of amines *via* C-H activation

Recycling of magnetic nano-ferrites catalyst for the α-cyanation of amines *via* C-H activation

XPS analysis of magnetic nano-ferrites

EDX analysis of magnetic nano-ferrites

XRD analysis of recycled magnetic nano-ferrites

SEM analysis of recycled magnetic nano-ferrites

XPS analysis of recycled magnetic nano-ferrites

^1^H and ^13^C NMR spectra of products

**General procedure for the synthesis of magnetic nano-ferrites**

3.47g of Ferrous sulfate (FeSO_4_·7H_2_O) and 5g of ferric sulfate Fe_2_(SO_4_)_3_ were dissolved in 130 mL water in a 500 mL beaker. Ammonium hydroxide (aq 25%) was added slowly to adjust the pH to 10. The reaction mixture was then continuously stirred for 5h at 50 °C. The ensuing nano-ferrites were separated magnetically, washed with deionized water until the pH reached 7, and then dried under vacuum at 50 °C for 12 h. The magnetic nano-ferrites were characterized by SEM, X-ray diffraction (XRD) and XPS analysis.

**General procedure for the synthesis of α-aminonitrile of amine *via* C-H activation**

Amine (1 mmol) was dissolved in 1:1 mixture of water and methanol, to which 25mg of the nano-ferrites was added and the contents were sonicated for 5 min. To this mixture, NaCN (1.1 mmol) and 30% of H_2_O_2_ (1 mmol) were added. The total contents were then introduced into the coil reactor (10 m) via peristaltic pump (under constant flow) at 0.8mL/min while maintaining the reaction zone temperature at 50 ^o^C. The product was then collected at the exit port and the catalyst extracted using an external magnet. The product was isolated by ethyl acetate extraction and purified using column chromatography and then characterized.

**Recycling of magnetic nano-ferrites catalyst for the α-cyanation of amines *via* C-H activation**

After completion of each reaction, the nano-ferrite catalyst was recovered using an external magnet, washed with water (100 mL), dried under vacuum at 50 °C (2h) and subsequently used for additional reactions. It was observed that catalytic activity was not diminished even after the sixth cycle.


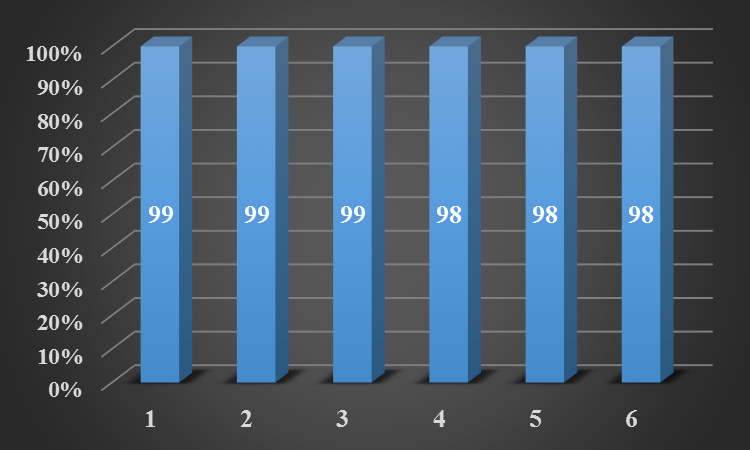


**S1:** Recycling of magnetic nano-ferrites


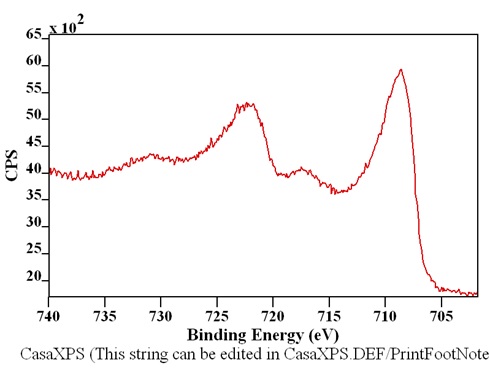
**XPS analysis of magnetic nano-ferrites**

**S2:** XPS analysis of magnetic nano-ferrites

**EDX analysis of magnetic nano-ferrites**

**S3:** EDX analysis of magnetic nano-ferrites


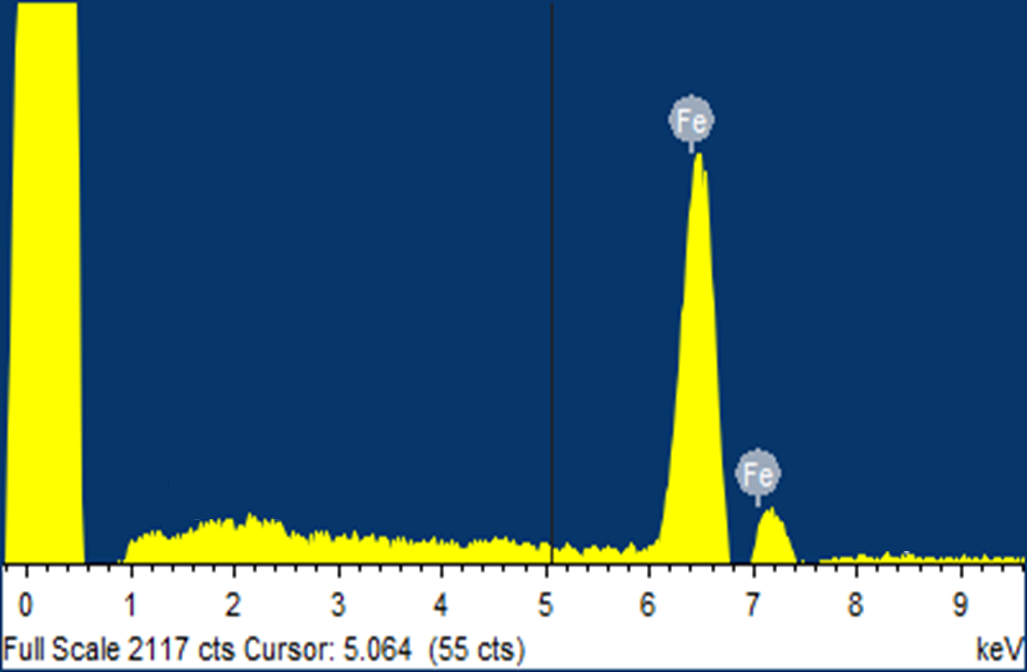


**SEM analysis of recycled magnetic nano-ferrites**

**
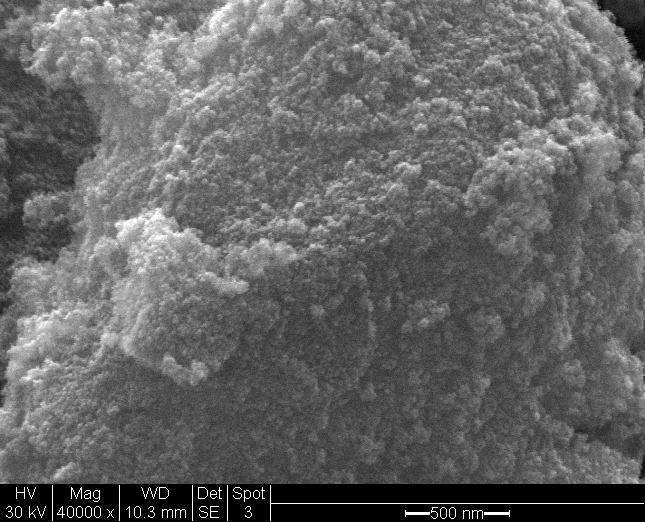
**

**S4:** SEM analysis of recycled magnetic nano-ferrites

**XPS analysis of recycled magnetic nano-ferrites**

**
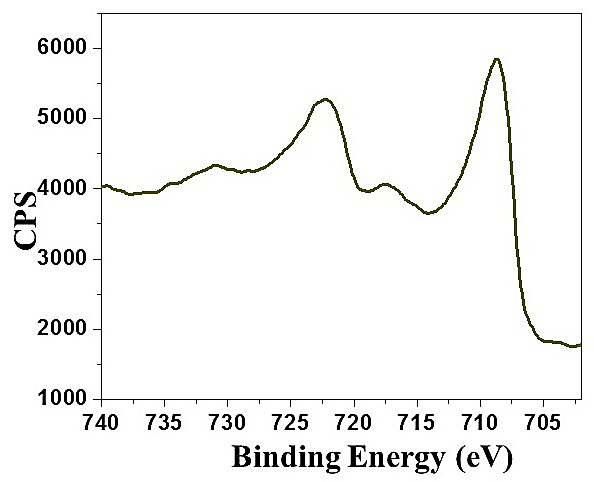
**

**S5:** XPS analysis of recycled magnetic nano-ferrites


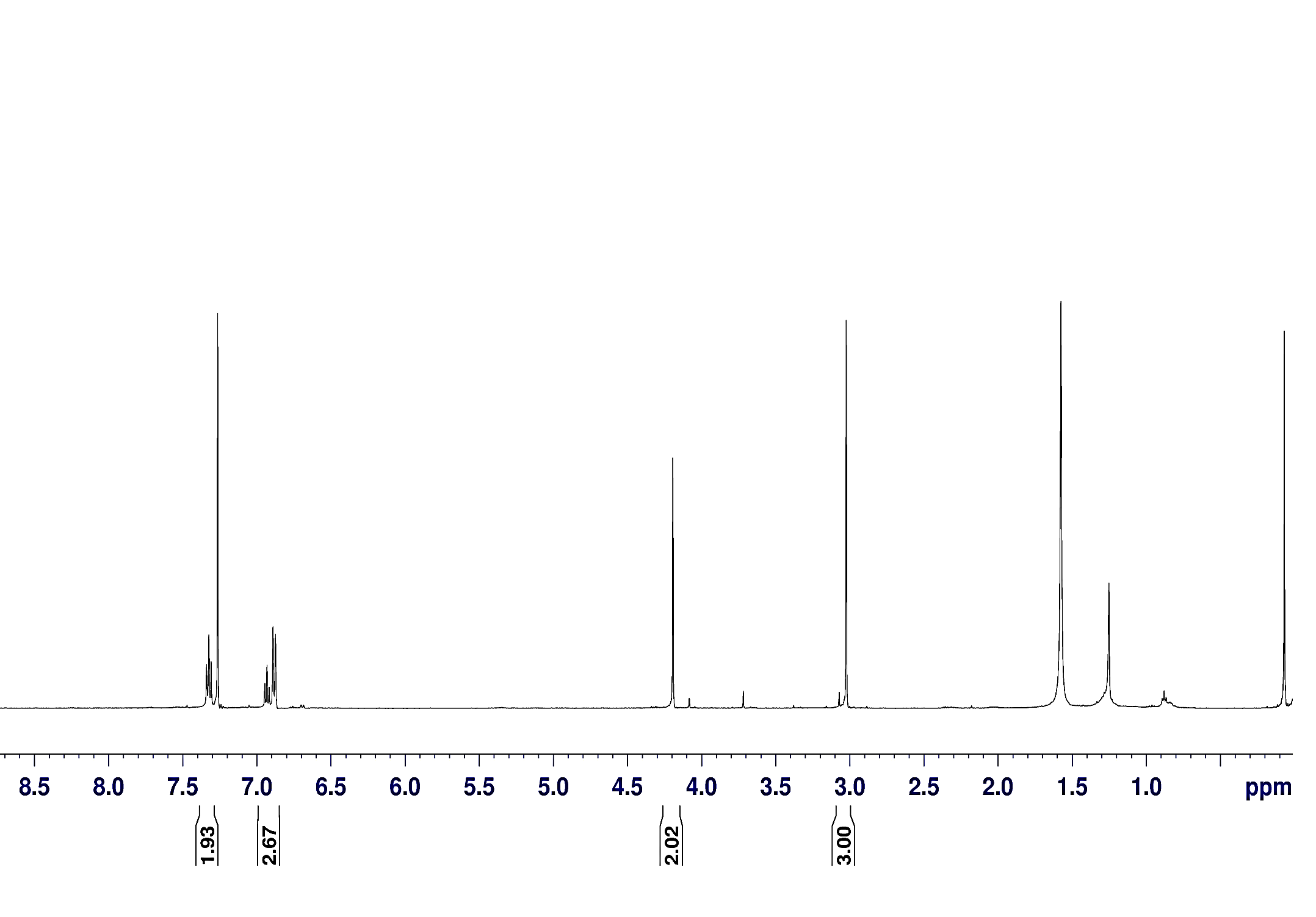


**S6:** ^1^HNMR for Table 2, entry 1


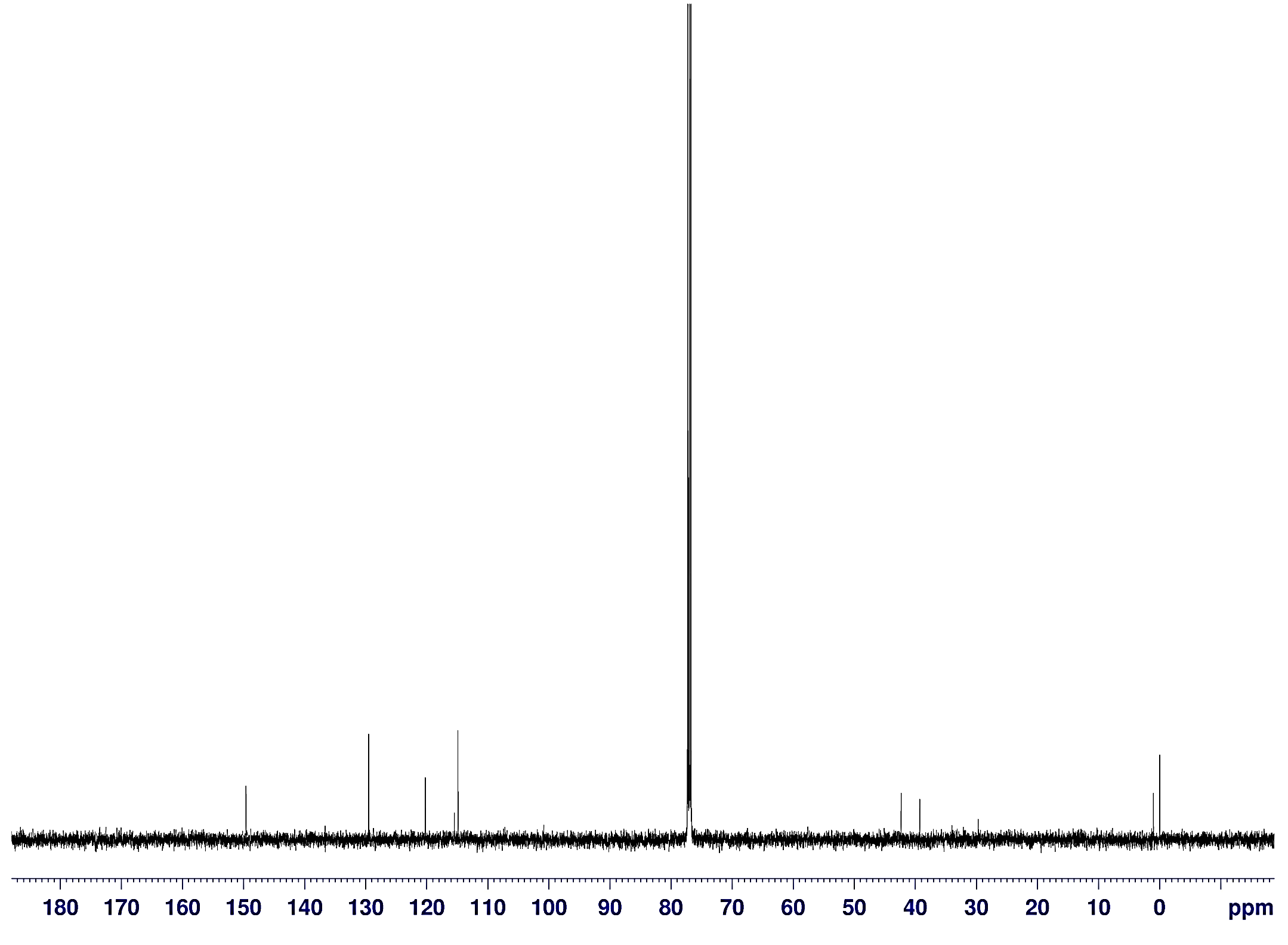


**S7:** ^13^CNMR for Table 2, entry 1


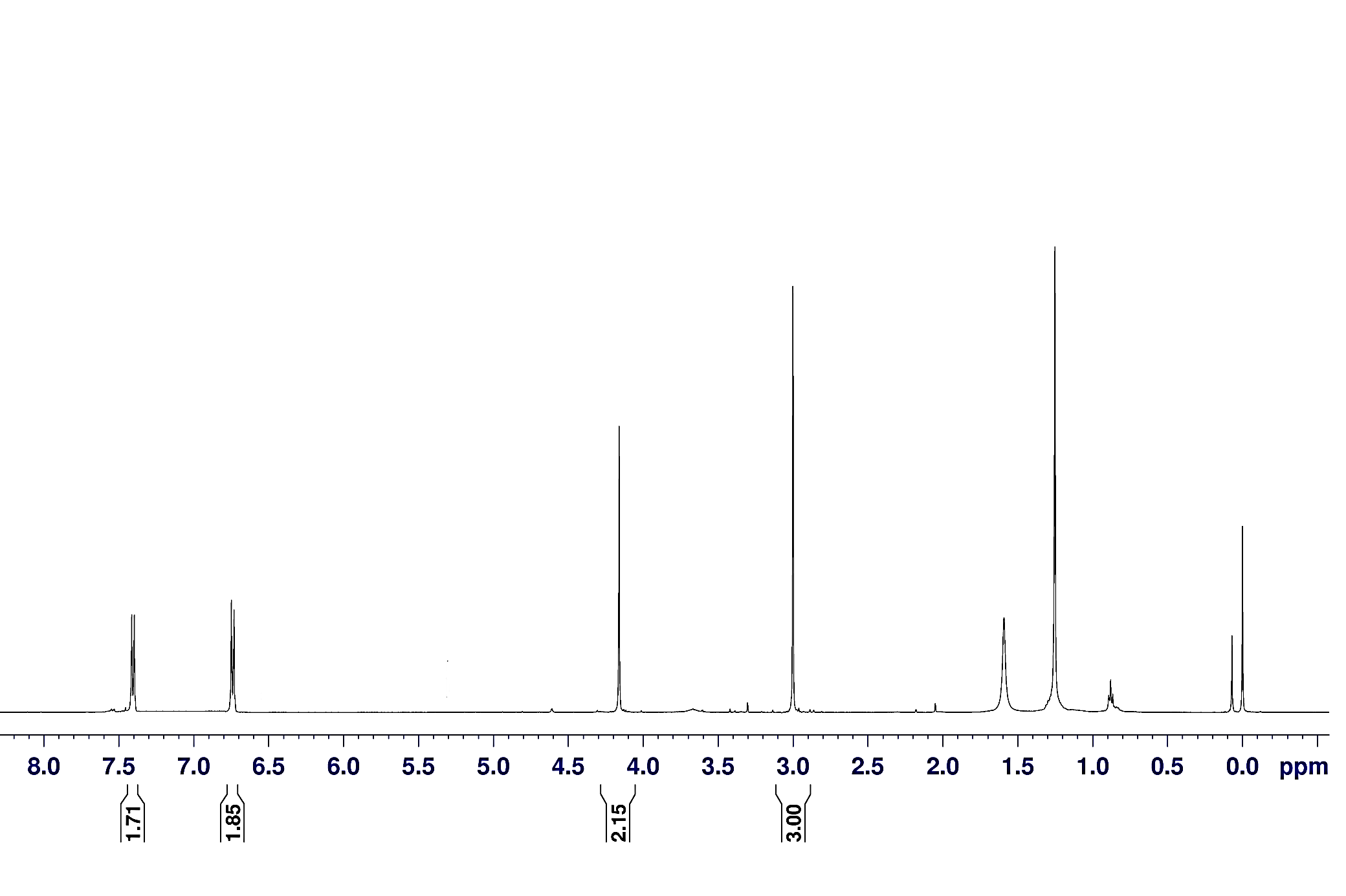


**S8:** ^1^HNMR for Table 2, entry 2


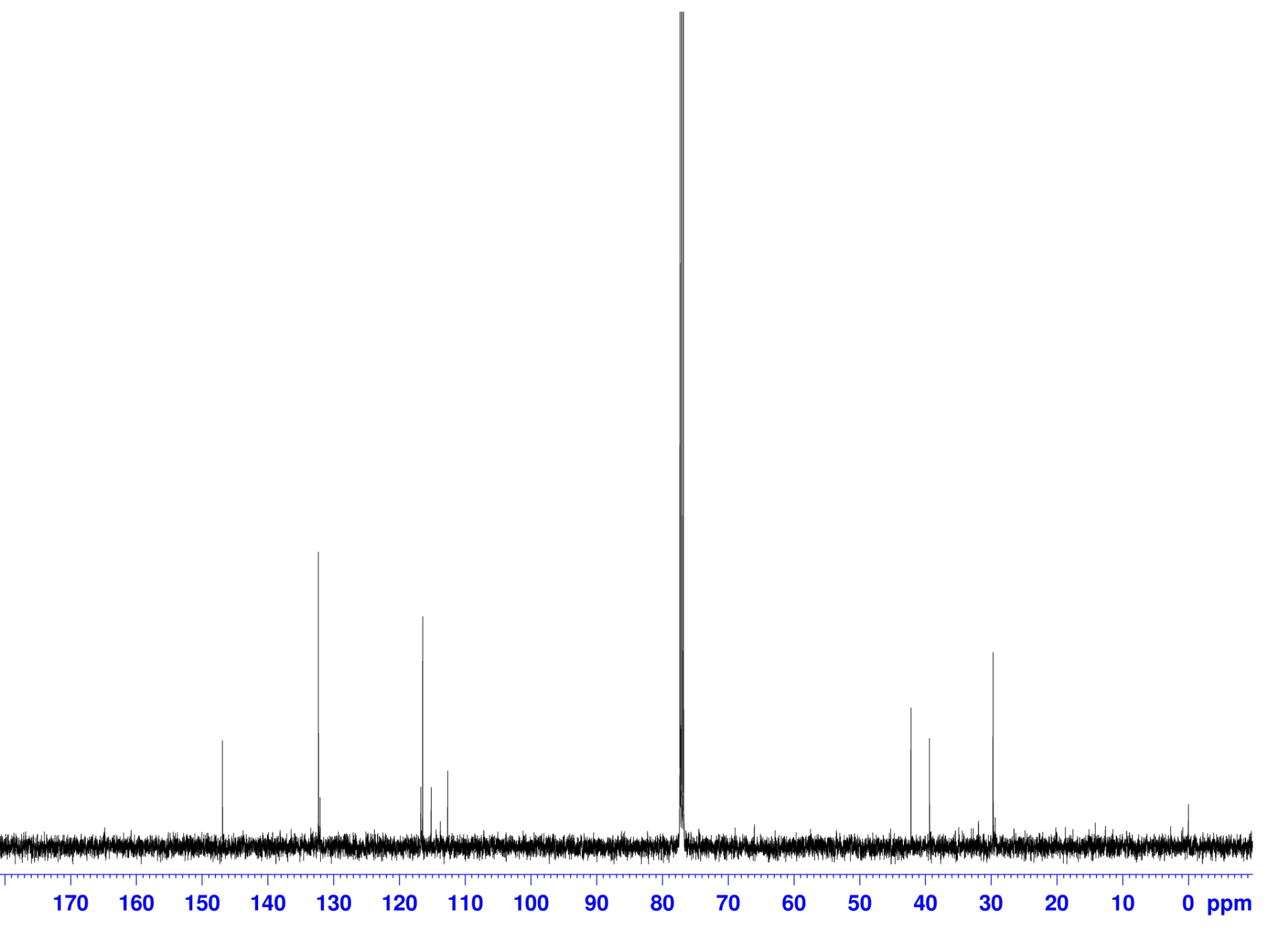


**S9:** ^13^CNMR for Table 2, entry 2


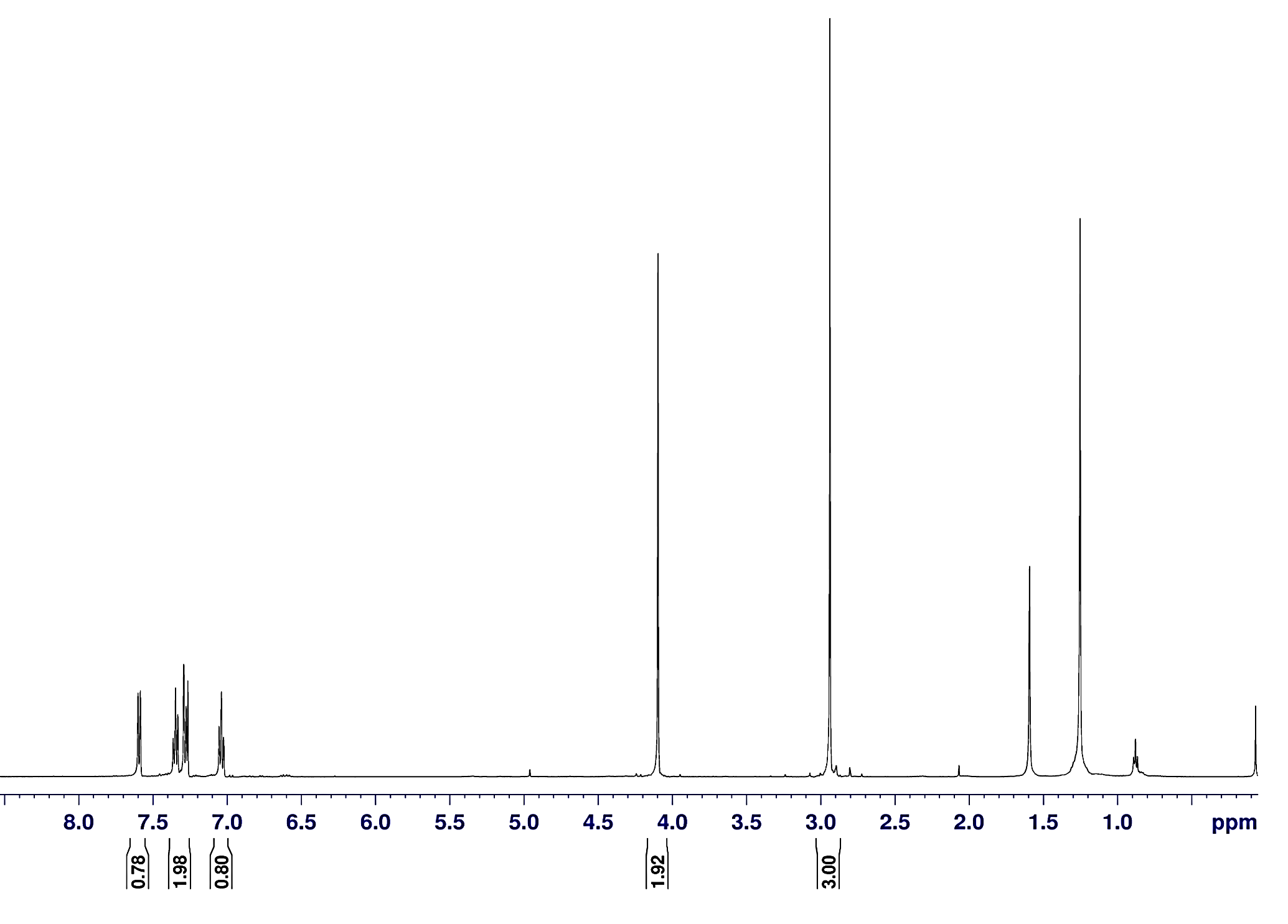


**S10:** ^1^HNMR for Table 2, entry 3


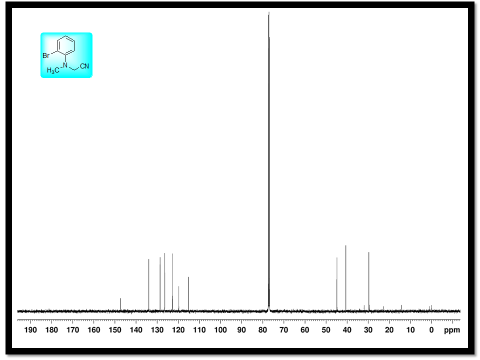


**S11:** ^13^CNMR for Table 2, entry 3





**S12:** ^1^HNMR for Table 2, entry 4


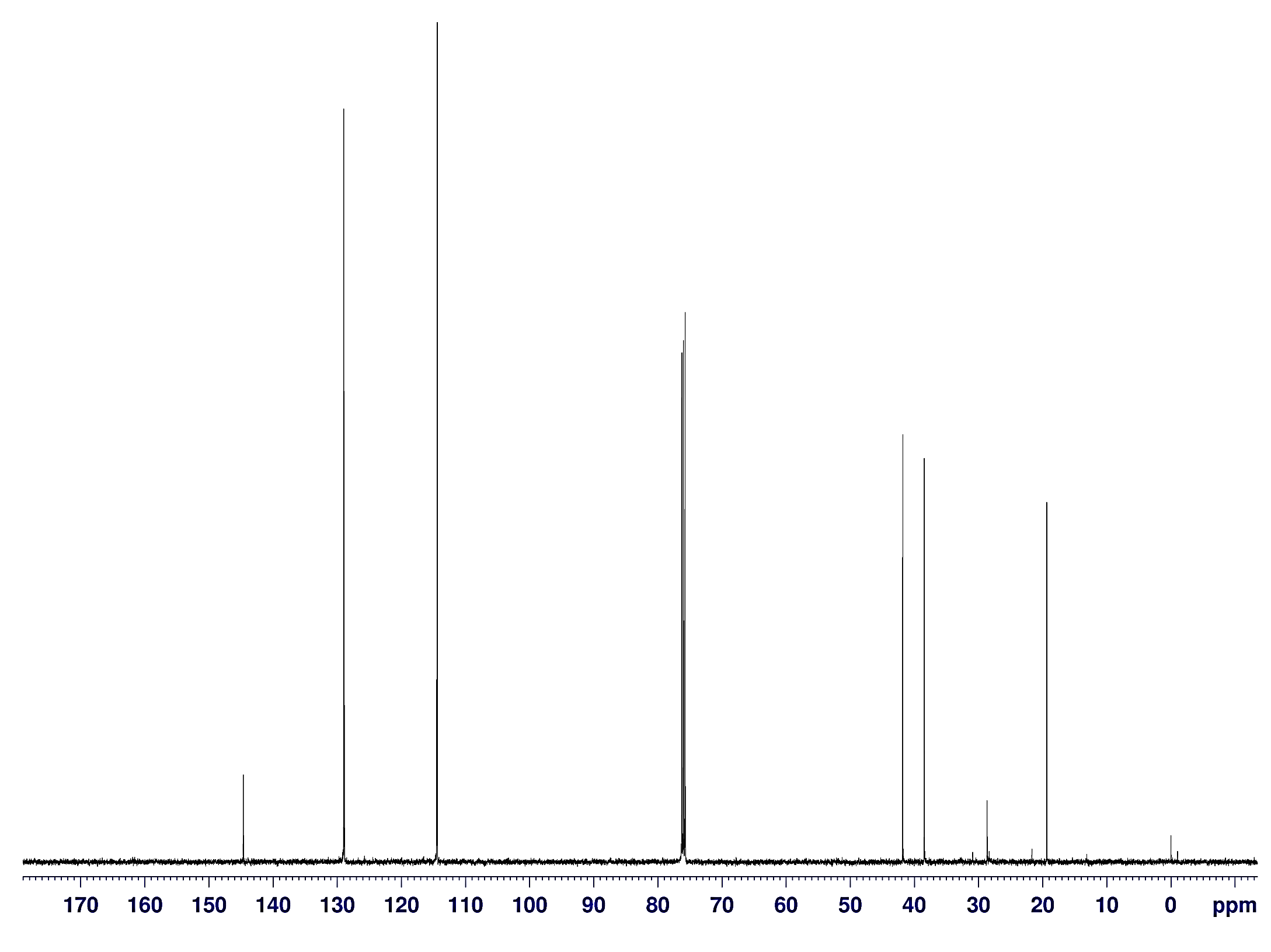


**S13:** ^13^CNMR for Table 2, entry 4


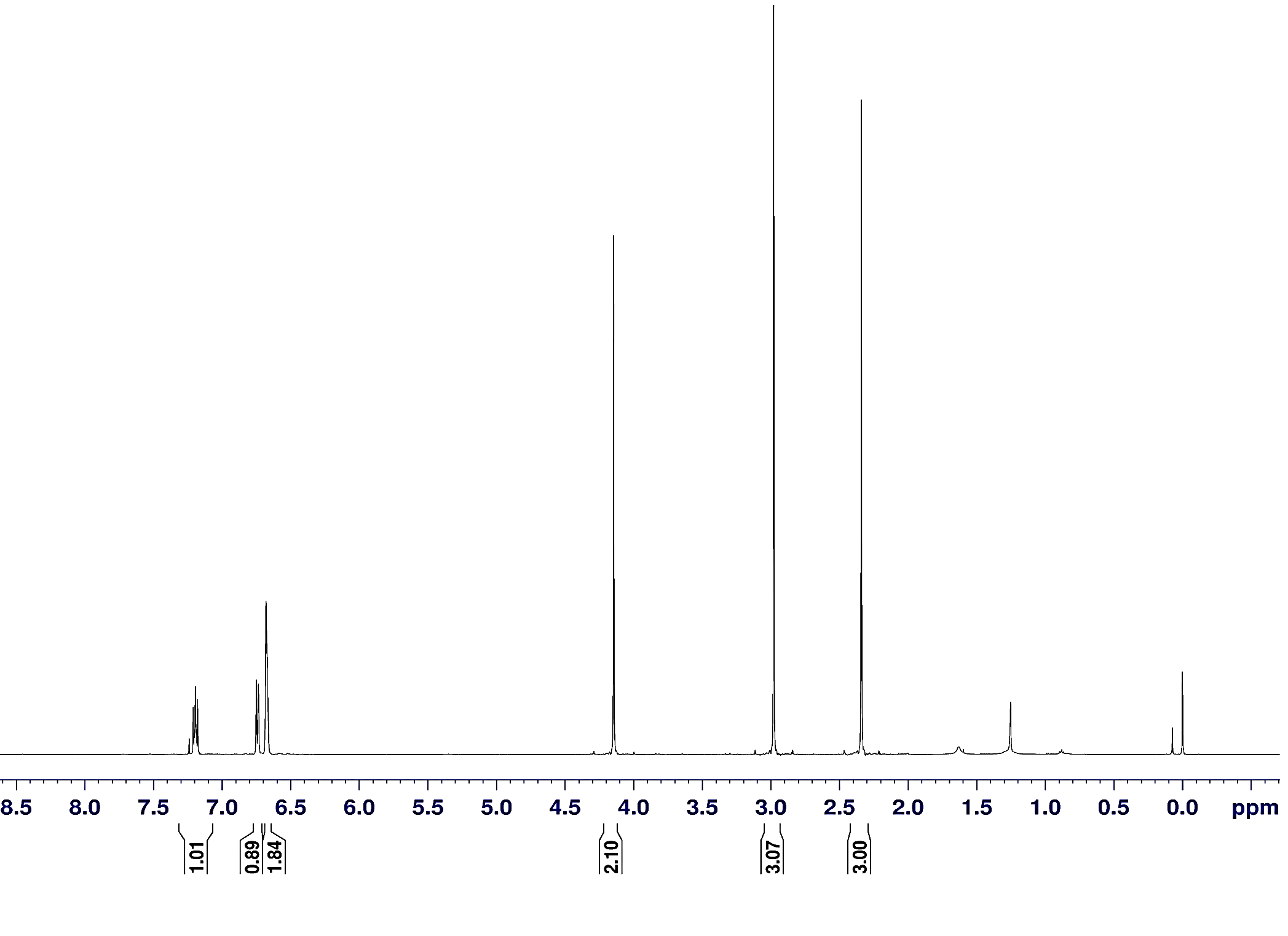


**S14:** ^1^HNMR for Table 2, entry 5


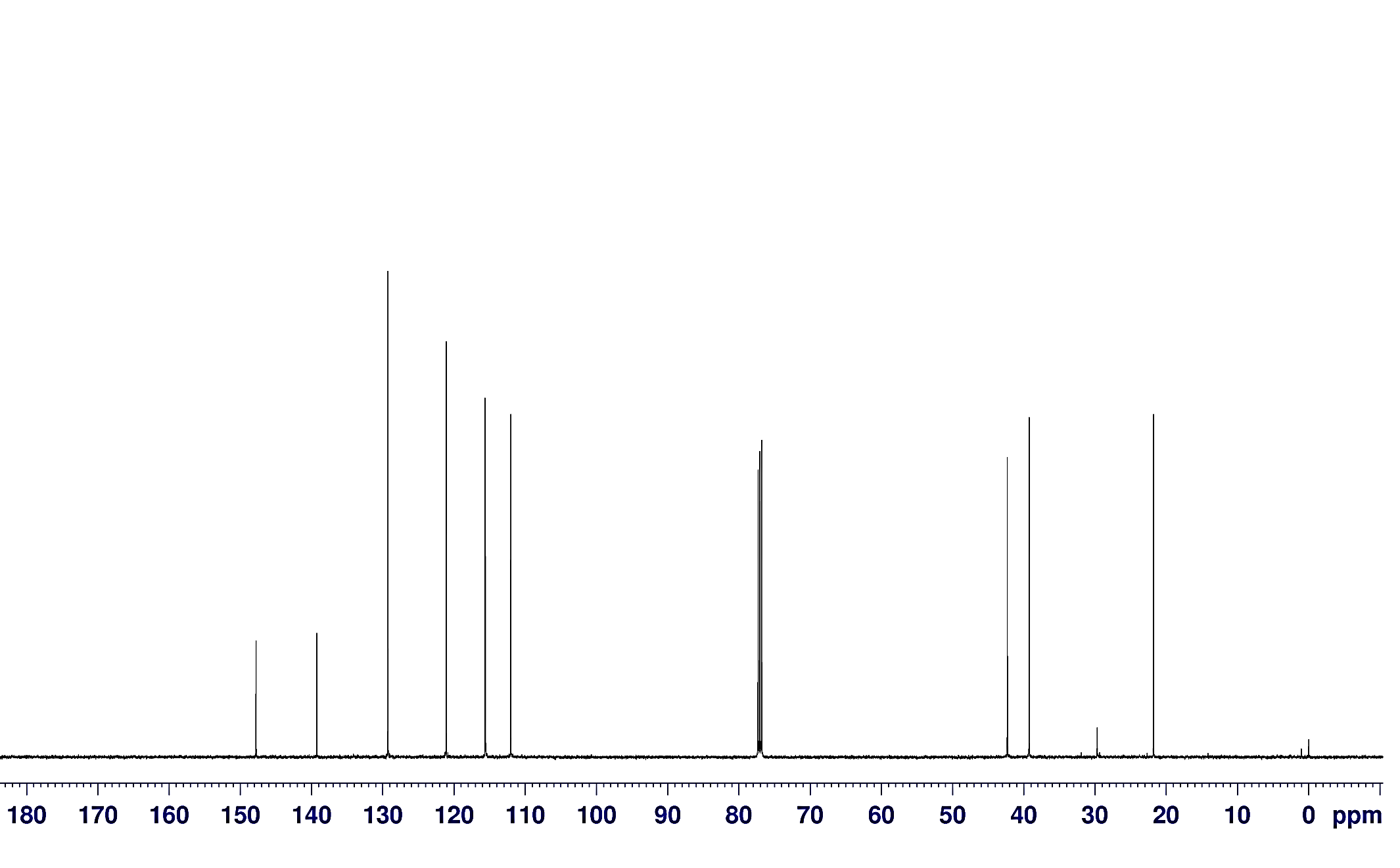


**S15:** ^13^CNMR for Table 2, entry 5


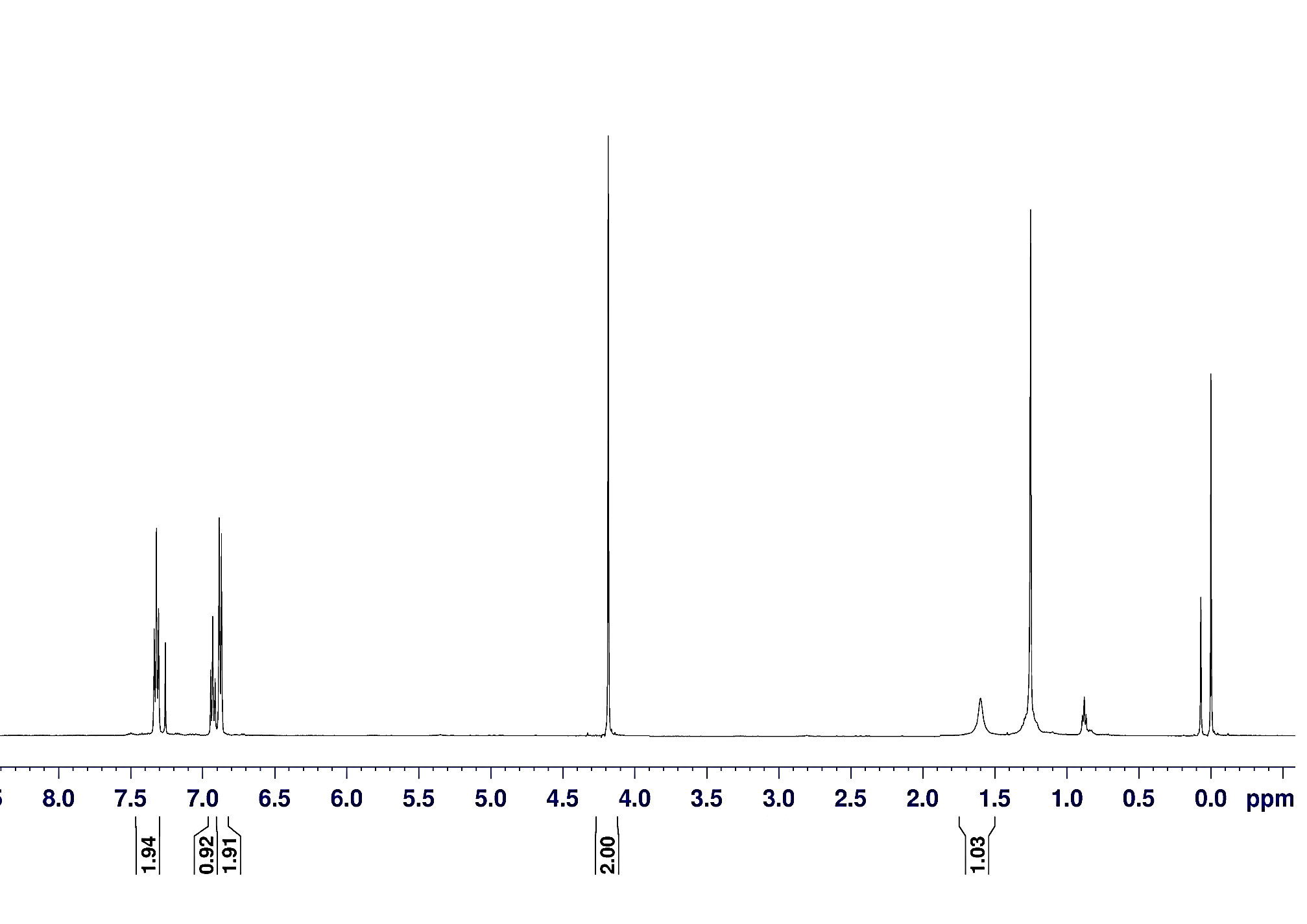


**S16:** ^1^HNMR for Table 2, entry 6


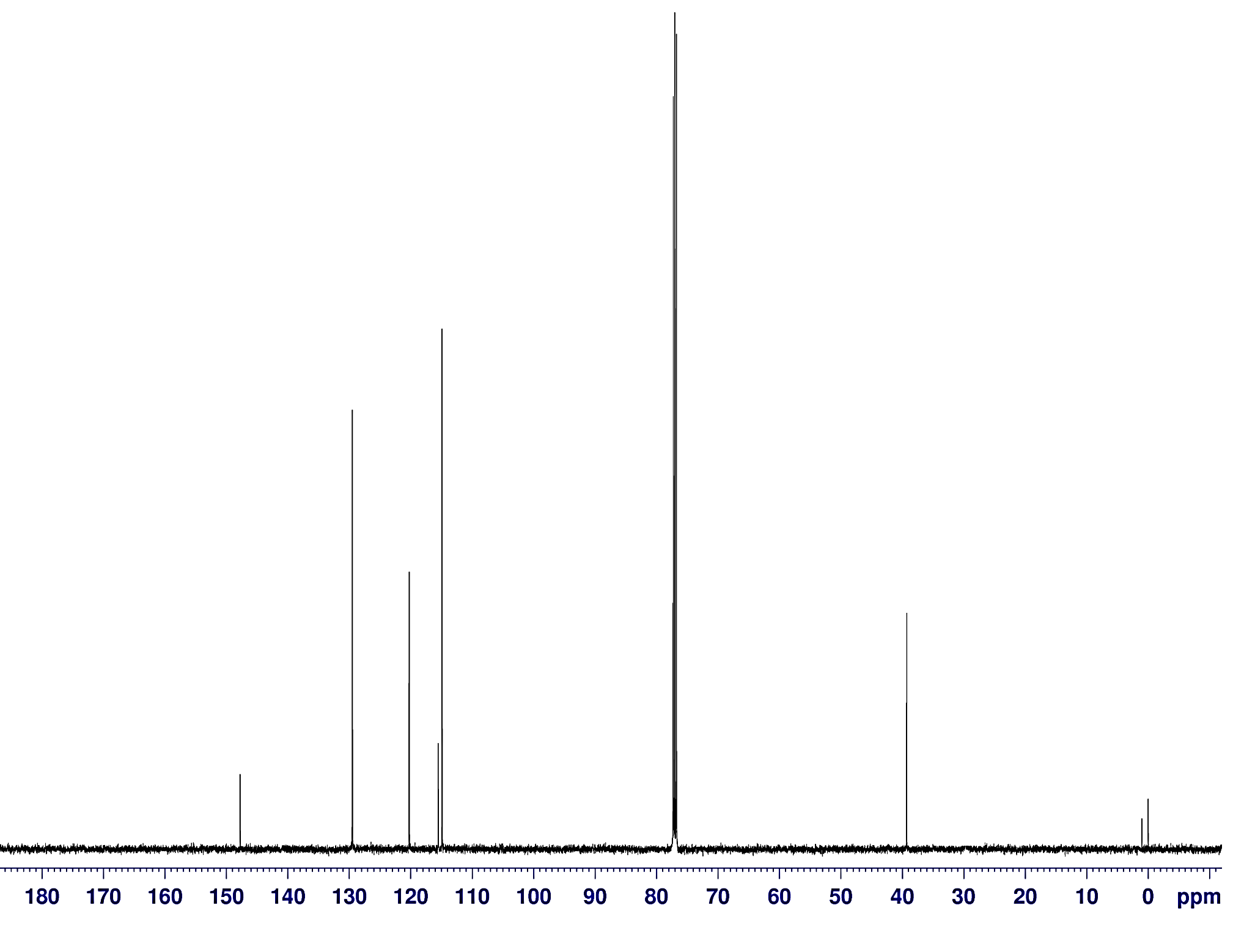


**S17:** ^13^CNMR for Table 2, entry 6
